# Stabilized single-injection inactivated polio vaccine elicits a strong neutralizing immune response

**DOI:** 10.1073/pnas.1720970115

**Published:** 2018-05-21

**Authors:** Stephany Y. Tzeng, Kevin J. McHugh, Adam M. Behrens, Sviatlana Rose, James L. Sugarman, Shiran Ferber, Robert Langer, Ana Jaklenec

**Affiliations:** ^a^David H. Koch Institute for Integrative Cancer Research, Massachusetts Institute of Technology, Cambridge, MA 02139;; ^b^Department of Biomedical Engineering, The Johns Hopkins University School of Medicine, Baltimore, MD 21231

**Keywords:** inactivated polio vaccine, vaccine stability, controlled release, global health, single-administration vaccines

## Abstract

Vaccination in the developing world is hampered by limited patient access, which prevents individuals from receiving the multiple injections necessary for protective immunity. Here, we developed an injectable microparticle formulation of the inactivated polio vaccine (IPV) that releases multiple pulses of stable antigen over time. To accomplish this, we established an IPV stabilization strategy using cationic polymers for pH modulation to enhance traditional small-molecule–based stabilization methods. We investigated the mechanism of this strategy and showed that it was broadly applicable to all three antigens in IPV. Our lead formulations released two bursts of IPV 1 month apart, mimicking a typical vaccination schedule in the developing world. One injection of the controlled-release formulations elicited a similar or better neutralizing response in rats, considered the correlate of protection in humans, than multiple injections of liquid vaccine. This single-administration vaccine strategy has the potential to improve vaccine coverage in the developing world.

## Significance

Inactivated polio vaccine (IPV) must be administered two to three times, with a 1–2 month gap between administrations, for patients to be protected. However, in the developing world healthcare workers often have difficulty reaching their patients multiple times to administer booster shots. We formulated IPV into microspheres that need to be injected only once and will be released in pulses with the desired timing without needing additional visits by a healthcare worker. To achieve this, we stabilized IPV using biocompatible excipients that allow it to remain in its active conformation inside the particles for months, and showed that they elicited a strong neutralizing immune response in rats, similar to that elicited by two separate injections of the traditional vaccine.

Because of the challenges of patient access in resource-poor settings, the World Health Organization (WHO) states that an ideal vaccine should be easy to administer with a single injection (1). Controlled release technologies that can release vaccine over time after a single injection could provide a potential solution for this problem. To achieve this, we sought to design a device that could: (*i*) encapsulate a vaccine that can be injected as a depot using a standard gauge needle and will remain in the patient for weeks or months; (*ii*) use a biocompatible material that degrades by hydrolysis to ensure consistent patient-to-patient release kinetics; (*iii*) stabilize the encapsulated vaccine against thermal and other physiological stresses over time; and (*iv*) release the vaccine in timed pulses that match the traditional vaccination schedule. We chose as the material poly(lactic-*co*-glycolic acid) (PLGA), a biodegradable polymer used for Food and Drug Administration-approved applications in humans and generally recognized as safe (2, 3). PLGA can encapsulate a vaccine and be injected into the patient as a degradable depot, releasing vaccine over time in pulses and providing the entire vaccination schedule with one injection. However, the use of PLGA-based systems for single-injection vaccines is hampered by numerous challenges, vaccine stability being the most prominent (4, 5). Many antigens and other proteins aggregate under conditions relevant to single-administration systems, including antigen drying, low pH, and incubation at body temperature (37 °C) (6–9).

One vaccine that could benefit from a single-administration delivery vehicle is the inactivated polio vaccine (IPV). Poliomyelitis is a potentially fatal but vaccine-preventable infectious disease. In many countries, IPV is administered in two to three injections of the liquid vaccine formulation, with 1 mo between each of the injections (10). While this is effective, it is infeasible in countries where patients may not have easy or regular access to healthcare. Studies in some of the relevant regions have found that many fewer children receive a second dose of vaccines than a first (11, 12), and children who receive only one injection of IPV remain unprotected from the disease, with seroconversion rates of as low as <20% after only one dose (10, 13). A single-administration vaccine could eliminate the need to return for a second injection and thereby protect patients, despite only one-time access to healthcare. This type of technology is of particular interest now due to the current efforts to eradicate polio, as successful eradication will require a very high coverage rate to ensure that the disease cannot be carried or transmitted by unvaccinated individuals. In many low-resource settings, an alternative to the traditional vaccination programs and schedules may be necessary for sufficient protection.

Additionally, because IPV is thermally unstable (14), excipients must be included in the formulation to maintain the D-antigen conformation of IPV, which correlates with protective immunogenicity (15, 16). We have previously used small-molecule excipients to protect IPV from thermal stress while encapsulated within microspheres (17). However, for this technology to be viable, the antigen must also remain stable despite the changing pH of the microsphere environment for the lifetime of the injected depot to induce neutralizing antibody production, which is required for protective immunity. In particular, aside from any intrinsic instability of the antigen’s conformation, the high-concentration, low-pH environment inside PLGA particles is well known to cause aggregation and denaturation of encapsulated proteins (18–20). While protein aggregation has been shown to induce an immune response in vivo (21), the high temperatures and pH variation that are observed in microparticles tend to cause the protein to aggregate in a nonnative conformation (22). As this type of aggregation is usually irreversible (23), it can lead to an antibody response that is not specific to the protective conformational epitope, or the D-antigen in the case of IPV, making the vaccine ineffective. We hypothesized that basic excipients would neutralize the acid formed by the degrading particles and that increasing the electrostatic repulsion among IPV virions would reduce or prevent their aggregation. Therefore, to improve antigen stability in the formulation, we selected three specific cationic polymers that are basic, cannot quickly diffuse out of the microspheres due to their size, and have a history of use as nanoparticle complexation agents. Throughout this report, we use the term “stability” to refer to the degree to which IPV retains its immunogenic (D-antigen) conformation as determined by ELISA.

We show here that the cationic polymer excipients Eudragit E, poly(l-lysine) (PLL), and branched polyethylenimine (bPEI) can be used to stabilize all of the three IPV antigens in PLGA microspheres. Having been used preclinically for drug delivery applications (24), cationic Eudragits have been shown to be safe and biocompatible (25); PLL, a polypeptide, is enzymatically degradable and is rapidly cleared from the body after administration (26); and bPEI, widely used for drug delivery, is nontoxic at the low molecular mass (1.8 kDa) used here (27). We designed PLGA-based microspheres with desirable release kinetics and high IPV stability. We then administered the IPV microspheres to rats in a single injection and compared their immunogenicity to that of a clinically relevant control of two boluses spaced 1 mo apart. To our knowledge, this report of a single-injection IPV formulation that could elicit a potent neutralizing immune response similar to that of multiple injections of a liquid bolus is unique. This indicates that our single-injection IPV formulation cannot only release stable D-antigen IPV over time in vitro but also provide protection in vivo, as the presence of neutralizing antibodies is considered the correlate of protection in humans for this vaccine (28, 29). Because the excipients studied here were used for their pH-modulating properties and electrostatic effects, we believe that they may potentially be applied broadly to stabilize many different vaccine antigens that normally aggregate under acidic conditions. This improved understanding of how excipients affect the pH environment and the physicochemical properties of antigens encapsulated in PLGA could open new directions for single-administration vaccine systems.

## Results

### Effect of Cationic Excipients on pH and PLGA Properties.

IPV can be encapsulated in PLGA to form F1 microspheres that release two bursts of IPV at 1 d and 25 d (17). This approximates the delivery of two human doses spaced 1 mo apart, mimicking a clinical vaccination schedule. However, the overall efficiency was low; only 5%, 6%, and 5% of the total loaded IPV types 1, 2, and 3, respectively, was released in D-antigen form (Table 1).

**Table 1.** Efficiency of D-antigen IPV release from microspheres

Particle formulation	Doses/mg particles (loaded) (%)	% D-antigen released (type 1)	% D-antigen released (type 2)	% D-antigen released (type 3)
F1	0.64	5	6	5
F2	0.64	17	56	20

Both microsphere formulations were loaded with the same initial amount of IPV, but the stabilizing properties of F2 allow much higher total release of IPV in its antigenic conformation.

**Fig. 1.**
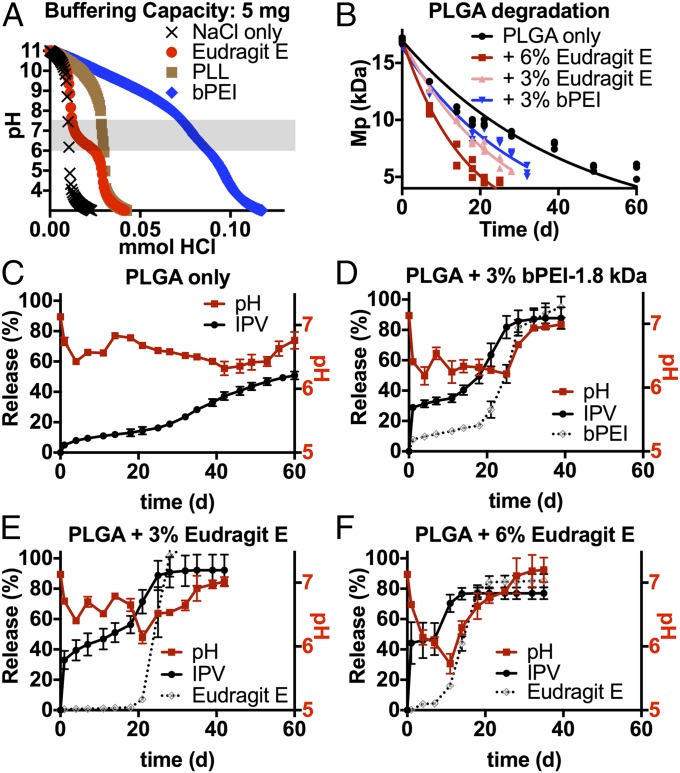
Eudragit E and bPEI have buffering capacity and affect PLGA degradation. The buffering capacity of Eudragit E, PLL, and bPEI was measured (*A*). After they were blended with PLGA and formulated into microspheres, Eudragit E and bPEI accelerated PLGA degradation over the course of a release study (*B*), observed as a difference in the rate at which the peak molecular weight (Mp) of the PLGA decreased. The release of acid from the microspheres into the external release medium correlated with PLGA degradation and total release of type 1 IPV, all of which are affected by incorporation of Eudragit E or bPEI (*C*–*F*). Release of Eudragit E or bPEI from the microspheres (plotted as a percentage of the total loading) was also pulsatile and matched the timing of IPV release. Data are reported as mean ± SD.

To develop a formulation with improved efficiency, we utilized various polycations that have been shown to be efficient at electrostatic complexation for biologics (16, 30), hypothesizing that their basic nature would counteract the build-up of acid within the degrading PLGA and thereby protect IPV from the decreasing pH. We measured the buffering capacity of the materials and focused on the pH range of interest, defined here as pH 6–7.4, outside of which the IPV D-antigen stability is dramatically reduced (17) (Fig. 1*A*). Because the loss of D-antigen is not reversed by neutralization of the pH, excipients that prevent the initial denaturation and aggregation events may be critical.

PLL had some buffering capacity at high pH, as expected due to its high pK_a_, but had little or no buffering capacity within the range conducive to IPV stability (Table 2), indicating that it was unlikely to play a major role in maintaining near-neutral microsphere pH. By contrast, Eudragit E had higher buffering strength in this range (2.31 mmol H^+^/g Eudragit E) than a standard neutral buffer like PBS (1.78 mmol H^+^/g PBS salts), indicating that this material may prevent excessive acidification in the PLGA environment. bPEI could buffer a similar amount of protons in the pH range of 6–7.4 but differs in that it is water-soluble; it can thus interact directly with the hydrophilic IPV.

**Table 2.** Buffering strength of polycationic excipients

Excipient or buffer	Protons buffered within pH 6–7.4 (mmol H^+^/g excipient or buffer)
None (NaCl only)	0
PBS	1.79
Eudragit E	2.31
bPEI	2.51
PLL	0.19

The number of protons buffered within the relevant pH range of 6–7.4 was calculated for each of the buffers or excipients relative to an unbuffered sodium chloride solution.

Although Eudragit E and bPEI were hypothesized to alleviate the acidification of PLGA particles that normally contributes to PLGA degradation by acid-catalyzed hydrolysis, the addition of the excipients was found to promote, rather than mitigate, accelerated PLGA degradation (Fig. 1*B*). Particles containing only PLGA, PLGA with 3% Eudragit E, PLGA with 6% Eudragit E, and PLGA with 3% bPEI had PLGA degradation half-lives of 29.6, 17.5, 11.5, and 21.6 d, respectively. This correlated with the kinetics of total IPV release from particles as well as the release of acid into the external release medium (Fig. 1 *C*–*F*), and the effect became stronger as the proportion of the excipients in the particle increased. Therefore, it is likely that Eudragit E and bPEI affect the local pH of the polymer phase in which they are blended, thereby causing accelerated PLGA degradation via base-catalyzed ester hydrolysis. This is also supported by the data collected on the pH of the release medium, which show that acid is released from the particle more quickly in the presence of these basic excipients, indicative of increased PLGA degradation. It is also noteworthy that the peak acidity of the release medium is observed very close to the time point at which the largest burst of IPV release is recorded. Eudragit E or bPEI is also released in a burst around the same time as IPV in these formulations (Fig. 1 *C*–*F*), further supporting the hypothesis that they act as buffers within the microspheres and help to facilitate IPV release upon their own dissolution and release.

### Effect of Cationic Excipients on IPV Stability and Physical Properties.

**Fig. 2.**
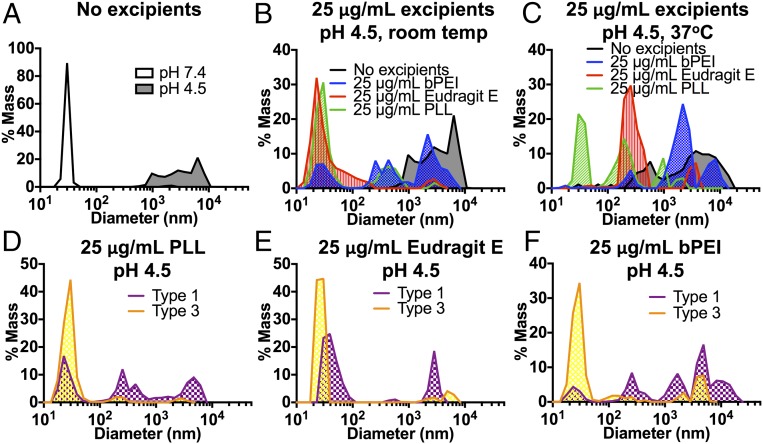
Acid-induced aggregation of IPV is prevented by complexation with polycations. IPV forms a broad distribution of large aggregates at low pH (*A*) when diluted in PBS without excipients. Cationic polymers, such as PLL, Eudragit E, and bPEI, prevent acid-induced aggregation at low pH (*B*). After 24 h of incubation at low pH, PLL is the only cation to preserve a large population of virus particles with diameters close to 30 nm at low concentration (25 μg/mL), while Eudragit E prevented some but not most of the aggregation (*C*). Serotypes 1 and 3 act differently in the presence of polycations, with type 3 showing the least aggregation at low pH in the presence of polycations (*D*–*F*).

In addition to their effect on PLGA, cationic polymers could also affect the properties of IPV. One of the mechanisms by which vaccines can lose immunogenicity is via aggregation (31), which can be exacerbated by changes in pH or extremely high concentrations when encapsulated in a delivery vehicle (32, 33). At a neutral pH of 7.4, dynamic light scattering (DLS) shows an IPV peak around 30 nm, the expected diameter of the virus particle (34). At low pH (pH 4.5), a condition relevant to PLGA-encapsulated materials, IPV can be seen to aggregate (Fig. 2*A*), as the peak shifts to the right and broadens, indicating the presence of IPV aggregates.

Polycations may, however, be able to affect the electrostatic properties of IPV, which has a net-negative surface charge, and prevent aggregation in solution. When trivalent IPV (tIPV) was mixed with cationic excipients before being diluted to pH 4.5, the net-positive charge conferred on the virus particles by the polycations seemed to prevent acid-induced aggregation by increasing the electrostatic repulsion among virus particles, as demonstrated by the much higher percentage of particles remaining in the 30-nm peak after complexation with PLL or Eudragit E (Fig. 2*B*). While the addition of bPEI seemed to preserve some of the 30-nm population of particles, it was far less effective than PLL and Eudragit E in preventing IPV aggregation at the same concentration (25 µg/mL). After 24 h of incubation at 37 °C, the differences among the cationic excipients became even more clear, as the addition of 25 µg/mL PLL preserved a substantial population of small particles (Fig. 2*C*). With 25 µg/mL Eudragit E, there was a large population of particles with a peak centered at 87.6 nm, suggesting that Eudragit E prevented the formation of large aggregates but not the formation of small ones.

The effect of IPV complexation with polycations was concentration-dependent. While 25 µg/mL PLL was able to prevent the formation of most IPV aggregates after 1 d of incubation at 37 °C at pH 4.5 (*SI Appendix*, Fig. S1*A*), a higher concentration (100 µg/mL) of Eudragit E and bPEI was able to prevent IPV aggregation nearly as well as PLL (Fig. 2 *D*–*F*). Moreover, the electrostatic interaction between polycations and IPV differed among the IPV serotypes. In particular, type 1 IPV was found to be more prone to aggregation than type 3, and the polycations had a much stronger positive effect on type 3 IPV particles than on type 1 (Fig. 2 *D*–*F*). This may indicate that the polycations could have a more significant effect on type 3 IPV than on the other serotypes, particularly for excipients like PLL that were found to be highly effective at preventing aggregation; nonetheless, aggregation could be prevented to some degree in all three serotypes using this method.

**Fig. 3.**
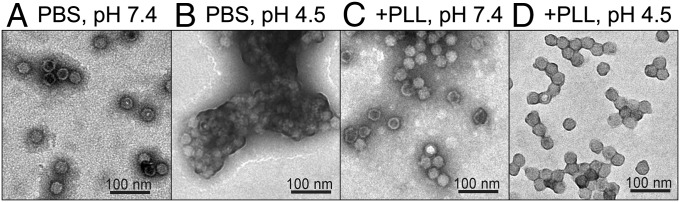
The polycation PLL mitigates acid-induced aggregation of IPV. IPV can be observed mostly as individual 30-nm particles at pH 7.4 (*A*) but forms massive aggregates at low pH 4.5 (*B*). Addition of PLL causes only a few minor aggregates at neutral pH (*C*) and prevents the formation of large aggregates upon acidification to pH 4.5 (*D*).

Transmission electron microscopy (TEM) imaging confirmed the effect measured by DLS. Individual IPV particles can be visualized at neutral pH with little to no observable aggregation (Fig. 3*A*). Very large aggregates form at pH 4.5 (Fig. 3*B*). The addition of PLL to IPV at neutral pH results in very few minor aggregates (Fig. 3*C*), and this precomplexation of IPV with PLL confers resistance to aggregation upon acidification to pH 4.5 (Fig. 3*D*).

### Effect of PLL and bPEI on IPV Release from Microspheres.

Because Eudragit E was a useful excipient that contributed to PLGA degradation, microsphere pH, and IPV stability in formulation F1, we examined the effects of alternatives to Eudragit E. During emulsification, bPEI was added to the organic phase to emulate the effect of Eudragit E on buffering the PLGA microenvironment and PLGA degradation, while PLL was added at low concentration to the aqueous phase to prevent excessive IPV aggregation at low pH. While bPEI has been incorporated into PLGA to complex with negatively charged cargo (35), to our knowledge, it has not previously been used specifically as an organic-miscible base to modulate the internal PLGA environment. Similarly, while PLL was chosen for its history of use as an electrostatic complexation material and as a method for controlling surface charge, its effect on IPV as a method of enhancing the stability of the vaccine has never before been reported.

**Fig. 4.**
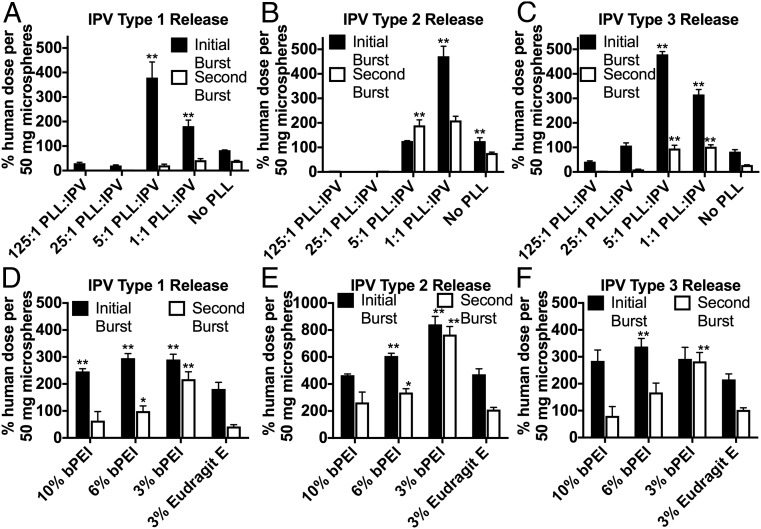
PLL and polyethylenimine promote stable IPV release from microspheres. All microspheres shown above contained in the same number of doses of tIPV and the same aqueous sugar- and salt-based excipients. Microspheres with Eudragit E cationic polymer in the organic phase released more stable IPV initially and in the second burst after addition of small amounts of PLL in the internal aqueous phase during emulsification, listed as molar ratio of PLL to IPV (*A*–*C*). Microspheres included PLL in the hydrophilic compartment and varying amounts of bPEI or Eudragit E in the organic polymer compartment. Microspheres containing 3–6% bPEI tended to release more stable IPV initially and in the second burst than those containing 3% Eudragit E (*D*–*F*). Groups that performed statistically significantly better than the control (“No PLL” in *A*–*C*; “3% Eudragit E” in *D*–*F*) are marked in the figure (*P < 0.05, **P < 0.01).

To first test the effect of PLL on IPV stability, particles based on F1 were fabricated, containing tIPV with aqueous excipients maltodextrin, monosodium glutamate (MSG), MgCl_2_, and PLL and organic excipient Eudragit E mixed with PLGA. The total D-antigen IPV release during the first and second burst was measured (Fig. 4 *A*–*C*), and 0.008–0.04% PLL loading (1:1–5:1 molar ratio of PLL:IPV) was found to significantly increase both the initial (0–4 d) and the later (20–40 d) IPV release in its D-antigen form. In particular, the initial burst of type 1 IPV was 2.2- and 4.5-fold higher after the addition of 1:1 and 5:1 PLL:IPV, respectively, while the initial burst of type 3 IPV was 3.8- and 5.8-fold higher, respectively. Although the initial type 2 release was also improved by the addition of 1:1 PLL:IPV, unlike for types 1 and 3, 5:1 PLL:IPV had no significant net effect on type 2 release (Fig. 4*B*). The addition of PLL also affected the second burst of IPV release. At these later time points, only 1:1 PLL:IPV had no negative effect on type 1 release; higher concentrations of PLL decreased the D-antigen IPV release from 20 to 40 d. However, both types 2 and 3 seem to have been stabilized significantly by the addition of 1:1 or 5:1 PLL:IPV, with 2.7- and 2.5-fold increases, respectively, of the second burst of type 2 IPV; and 3.6- and 3.4-fold increases, respectively, of the second burst of type 3 IPV (Fig. 4*C*). Because 1:1 PLL:IPV had a significantly positive effect and no significant negative effect on the D-antigen release of all three IPV serotypes both initially and during the second burst, this amount of PLL was incorporated into the aqueous phase of the microsphere emulsion for further testing.

Then, microspheres were formulated with 1:1 PLL:IPV in the inner aqueous phase of the emulsion along with tIPV and other excipients and either 3% Eudragit E or varying amounts of bPEI in the organic polymer phase (Fig. 4 *D*–*F*). The initial and second bursts of IPV release from the resulting microspheres were measured again. Generally, use of bPEI led to D-antigen IPV release similar to or greater than the IPV release from particles with 3% Eudragit E. For the initial burst, replacing 3% Eudragit E with 3%, 6%, or 10% bPEI resulted in 1.4-, 1.6-, or 1.6-fold higher type 1 IPV release, respectively; statistically similar and 1.3- or 1.8-fold higher type 2 IPV release, respectively; and 1.3-, 1.6-, or 1.4-fold higher type 3 IPV release, respectively. As with PLL, a greater effect was seen in the second burst of release, with 5.1-, 3.6-, and 2.8-fold higher release of stable types 1, 2, and 3 IPV when 3% bPEI was used.

**Fig. 5.**
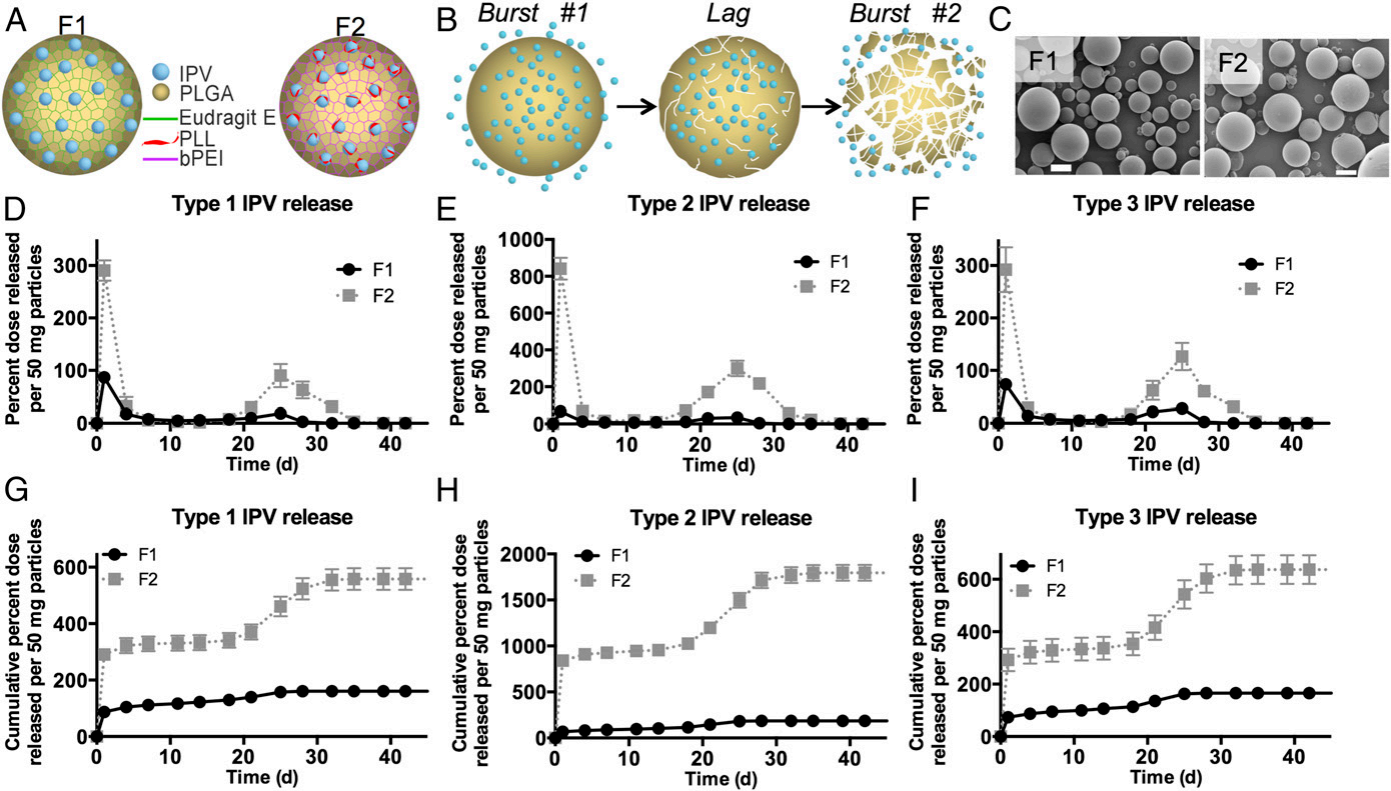
F1 and F2 microspheres release stable IPV over >3 wk. F1 and F2 microspheres contain Eudragit E, PLL, and/or bPEI as excipients, as depicted in the schematic (*A*). As depicted in *B*, the initial burst of release results from diffusion of IPV on or near the surface of the particles, followed by a lag phase as PLGA begins to degrade by chain scission and finally a second burst as degradation proceeds enough for the particle to lose mass and release internally encapsulated cargo. F1 and F2 microspheres have smooth morphology by SEM (*C*). (Scale bar, 20 µm.) Release (*D*–*F*) and cumulative release (*G*–*I*) graphs show that all three IPV serotypes are released in D-antigen conformation in two distinct bursts and that F2 microspheres deliver more stable IPV than F1.

Accordingly, for our new formulation, F2, we replaced Eudragit E with PLL, mixed 1:1 with IPV in the internal aqueous phase during emulsification to promote direct interaction with the IPV, and 3% bPEI in the organic polymer phase for close interaction with the PLGA (Fig. 5 *A* and *B*). Scanning electron microscopy (SEM) and sizing by Coulter Counter showed that the F2 microspheres were spherical and smooth with an average diameter of 10.5 ± 2.8 µm, similar in morphology to the F1 microspheres, which have an average diameter of 11.2 ± 3.4 µm (Fig. 5*C*). The release and cumulative release graphs of F2 (Fig. 5 *D*–*I*) show release of IPV between 20 and 30 d, but while the second burst of release from 50 mg F1 microspheres was 31%, 70%, and 52% of a human dose of serotypes 1, 2, and 3, respectively, the same amount of F2 microspheres released 218%, 769%, and 283% of a human dose in the second burst. Additionally, a high total amount of D-antigen IPV was released over the course of the study. While only 5%, 6%, and 5% of the total loaded amounts of types 1, 2, and 3 D-antigen IPV, respectively, was released from formulation F1, F2 released 17%, 56%, and 20% of types 1, 2, and 3, respectively, suggesting better stability of the encapsulated IPV (Table 1).

### Immunogenicity of Eudragit E-Doped F1 Microspheres.

**Fig. 6.**
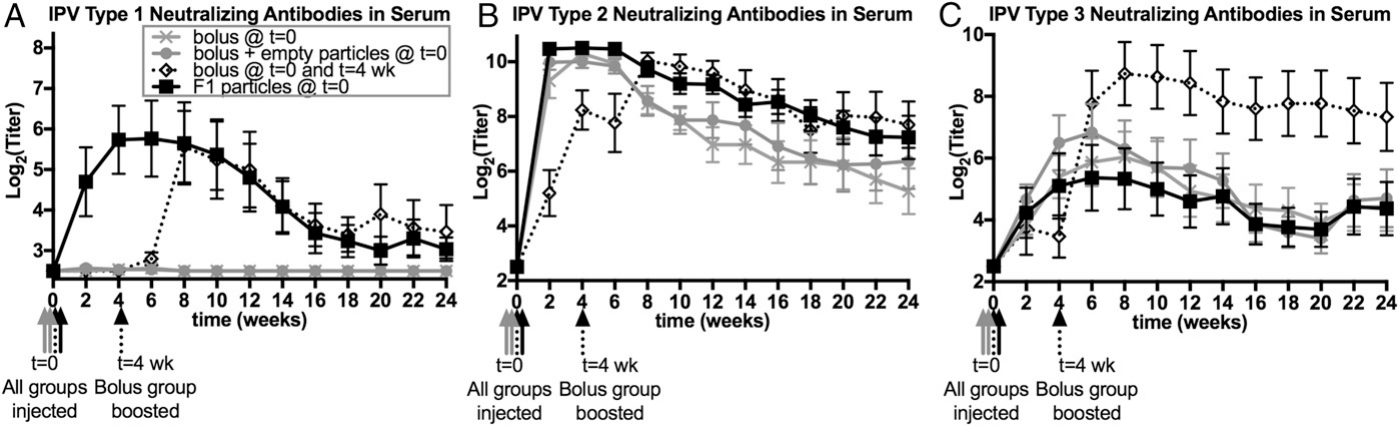
F1 microspheres elicit a strong neutralizing response against type 1 and type 2 poliovirus. The neutralizing antibodies in the serum of immunized rats is shown as the geometric mean absolute titer (*A*–*C*) for types 1, 2, and 3, respectively. A single bolus injection of liquid IPV is represented by the gray X; a single bolus injection of liquid IPV along with empty microspheres is represented by closed gray circles; two bolus injections of liquid IPV at *t* = 0 and 4 wk is represented by open black diamonds; and a single injection of F1 microspheres is represented by closed black squares. Data represent geometric mean ± geometric SE.

The neutralizing response of rats immunized with boluses of liquid IPV or a single injection of F1 or F2 microspheres is reported as absolute antibody titers (Fig. 6 and *SI Appendix*, Fig. S2). Throughout this report, “noninferiority” of neutralizing antibody titers is defined as titers superior to the multiple-bolus control or not statistically different from the control with a confidence interval of 95%. For type 1 IPV, no neutralizing response was seen after a single bolus injection of liquid vaccine (Fig. 6*A*). Only after a second bolus injection 1 mo later were neutralizing antibodies detected, with a geometric mean titer of 7.0 ± 1.4 [log_2_(titer): 2.8 ± 0.5] after 2 wk. Titers peaked 4 wk after the boost, with a geometric mean titer of 47.4 ± 7.0 [log_2_(titer): 5.6 ± 2.8]. In contrast, F1 microspheres containing the same dose of D-antigen IPV required only a single injection to elicit high neutralizing titers [26.0 ± 6.5 geometric mean, log_2_(titer): 4.7 ± 2.7] within 2 wk, which peaked at 4 wk [53.2 ± 6.3 geometric mean, log_2_(titer): 5.7 ± 2.6]. Importantly, even at late time points, F1 microspheres elicited a neutralizing response that was noninferior to that induced by the clinical control of two separate bolus injections, which may indicate that long-lived immunity is possible using this approach.

Type 2 IPV had a similar effect, with high neutralizing titers within 2 wk after injection. It was clear that the magnitude of the neutralizing response to a single injection of liquid IPV was dose-dependent, with a single injection of the full dose of a liquid IPV bolus (4.8 DU) eliciting a stronger response at early time points than a single injection of half the dose (2.4 DU) (Fig. 6*B*); however, after the latter group received a booster injection at 4 wk with the second half of the dose (2.4 DU), the titers increased substantially and were maintained for a longer time. Interestingly, F1 microspheres elicited a stronger initial response than the highest dose of a single bolus at 2 and 4 wk and also maintained high titers for as long as the two-bolus control at late time points.

In contrast to type 1 and 2, the type 3 IPV released from F1 microspheres elicited a significantly weaker response in vivo than the two-bolus control of liquid IPV (Fig. 6*C*). Therefore, F2 microspheres, formulated with the PLL and bPEI that were shown to be beneficial to in vitro IPV stability, particularly for type 3, were also tested in the in vivo rat model.

### Immunogenicity of F2 IPV Microspheres.

**Fig. 7.**
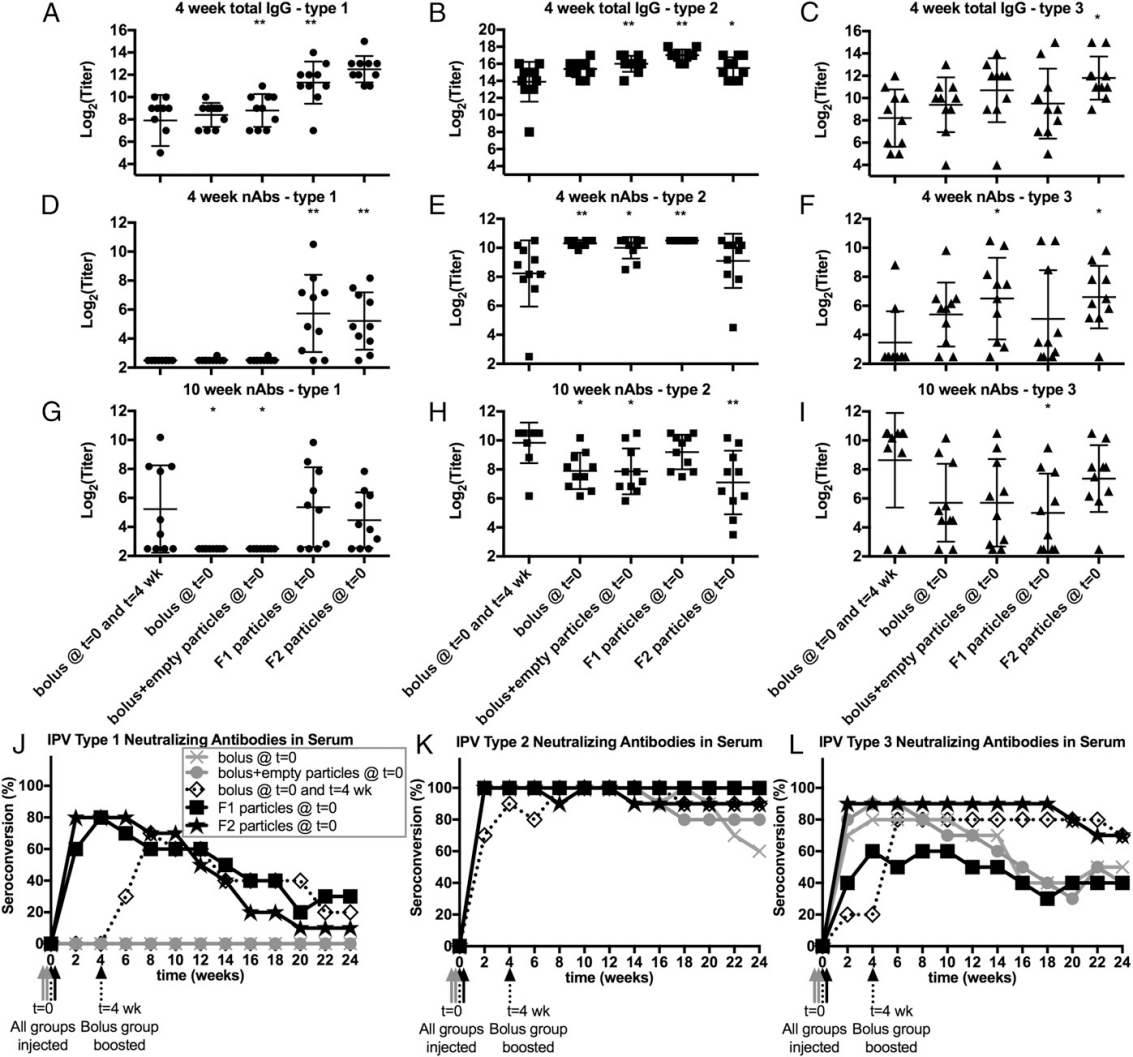
Formulations F1 and F2 combined elicit a strong neutralizing antibody response to all three IPV serotypes. The total IgG binding titers against IPV type 1 (*A*), 2 (*B*), and 3 (*C*) are shown 4 wk after injection. The absolute neutralizing antibody titers against poliovirus types 1 (*D* and *G*), 2 (*E* and *H*), and 3 (*F* and *I*) elicited by the bolus controls and formulations F1 and F2 are shown before (*D*–*F*) and after (*G*–*I*) the bolus control group received its second injection. Seroconversion (the percent of animals with detectable neutralizing antibody titers) was comparable in rats immunized with a single injection of F1 or F2 microspheres as in rats administered two standard injections of the liquid bolus (*J*–*L*). Asterisks indicate statistically significant differences (*P < 0.05, **P < 0.01) compared with the control (bolus injected at *t* = 0 and *t* = 4 wk) at the time point shown.

F2 microspheres were injected into rats, and the total IgG response and the neutralizing antibody response were both measured. After 4 wk, a single bolus injection of liquid IPV elicited an IgG response to all three serotypes (Fig. 7 *A*–*C*), but the response to IPV types 1 and 2 was significantly stronger when encapsulated in F1 microspheres (*P* < 0.01), and the response to all three IPV serotypes was significantly stronger when encapsulated in F2 microspheres. For types 1 and 2, coinjection of empty PLGA microspheres alongside liquid IPV also causes a lower but still statistically significant increase in total IgG titers. As seen in Figs. 6*A* and 7*D*, however, the antibodies raised against a single bolus injection of liquid type 1 IPV were not neutralizing. In contrast, one injection of formulation F2, like F1, was sufficient for high seroconversion, defined as the percentage of animals in a group with detectable neutralizing antibodies. F2 elicited detectable neutralizing antibodies against type 1 poliovirus in 80% of all tested animals within 4 wk (Fig. 7 *D* and *J*). Interestingly, although type 2 IPV was the easiest to stabilize in vitro and formulation F1 elicited a strong type 2 neutralizing response in vivo, an injection of F2 resulted in 100% seroconversion but lower absolute neutralizing antibody titers than the previous formulation F1 (Fig. 7 *E* and *K*). As expected, due to the higher in vitro stability, formulation F2 had the greatest effect on the type 3 neutralizing response in vivo, with 90% seroconversion within the first 4 wk and higher absolute antibody titers than any of the other tested groups, including the F1 microsphere group (Fig. 7 *F* and *L*). In the liquid bolus control group, a second injection caused increased neutralizing antibody production, as expected, but even then the neutralizing antibody response to type 3 poliovirus elicited by the bolus control group was not statistically significantly different from that elicited by a single injection of formulation F2 (Fig. 7*I*).

For type 1 IPV, the seroconversion rate peaked at 70% after two injections of a liquid bolus and at 80% after injection of either formulation F1 or F2 (Fig. 7*J*). The portion of animals still seropositive for type 1 neutralizing antibodies after 24 wk was 20%, 30%, and 10% for the bolus control, F1, and F2, respectively. For type 2 IPV, 100% of the animals had seroconverted at the peak of the response, and seroconversion at 24 wk was 90%, 100%, and 90% for the control, F1, and F2, respectively (Fig. 7*K*). For type 3 IPV, the peak response to the control, F1, and F2 was 80%, 60%, and 90% seroconversion, respectively, with 70%, 40%, and 70% seroconversion at 24 wk, respectively (Fig. 7*L*). Therefore, formulation F1 was noninferior to the two-bolus control for IPV types 1 and 2, while formulation F2 was noninferior to the two-bolus control for IPV type 3.

## Discussion

The development of single-administration IPV formulations, which could potentially have a significant impact on vaccine coverage and seroprotection against polio in resource-poor settings, has been hampered by the instability of the vaccine under physiologically relevant conditions. While some groups have reported stabilization of IPV in solid form for thermostability during storage (36) or dose reduction (37–40), these studies do not aim to reduce the number of repeated injections required for protective immunity. Here, we show that IPV in PLGA-based microspheres can be stabilized with small-molecule excipients and also protected against changes in pH by interactions with polycations. The resulting pulsatile-release formulations can elicit a neutralizing immune response noninferior to that of two bolus injections at clinically relevant concentrations. We also demonstrate general principles by which electrostatic complexation can be used to achieve antigen stability in single-injection PLGA systems.

The immune response to type 3 IPV in F1 microspheres was not as strong as the response to the other two components, types 1 and 2, demonstrating the importance of optimizing parameters for each immunogen in a vaccine formulation. We and other groups have previously reported significant differences in the D-antigen stability of the various serotypes (17, 36), which may contribute to the differences in the in vivo immune response to each. One distinction between type 3 IPV and the other two serotypes is its relatively low isoelectric point (pI), which has been reported to be ∼7.0–7.1 for Brünhilde strain type 1 (41, 42), 6.7–6.8 for MEF-1 strain type 2 (42, 43), and 5.8 for Saukett strain type 3 (43). Upon reaching this pH, the normally negative IPV virus particles lose their net charge, allowing aggregation to occur. We hypothesized that the lower pI would make interactions with cationic excipients more significant for type 3 than for types 1 and 2. Thus, Eudragit E was examined in detail, along with other cationic polymers.

As seen in Figs. 1 and 2, Eudragit E, an important component of formulation F1, performs multiple functions in the microspheres. As an organic-soluble base, it can be blended into the PLGA phase. Rather than only buffering acidic protons resulting from PLGA ester hydrolysis, which would result in slower degradation as the pH is kept near neutral, Eudragit E accelerates PLGA ester hydrolysis by locally increasing the pH and facilitating base-catalyzed PLGA degradation. This accelerated bulk PLGA erosion results in a sudden release of IPV days or weeks after the initial burst (Fig. 1), allowing the formulation to better mimic a clinical vaccination schedule of two bolus injections. Triphasic release kinetics have commonly been reported for PLGA and other bulk-eroding polymers (44), and the addition of excipients that further accelerate internal degradation emphasizes this effect, leading to pulsatile release with kinetics that can be tailored by changing the amount of basic excipients, as we have previously reported (17). Moreover, the timing of the IPV release is correlated with the timing of sudden pH changes and Eudragit E release, suggesting that Eudragit E becomes successively more protonated until it becomes soluble in the low-pH environment and diffuses away, further increasing the particle porosity. The timing of both the pH and the IPV release peaks can be adjusted depending on the amount of Eudragit E incorporated into the particles, providing an additional method of modulating vaccine-delivery kinetics.

Eudragit E may also have the additional benefit of preventing IPV aggregation at low pH. Although Eudragit E is water-insoluble at neutral pH, it becomes increasingly soluble as the pH inside the microspheres decreases, allowing it to partition more into the hydrophilic microsphere compartment to associate with IPV. Thus, the decrease in pH as PLGA degrades, which would otherwise cause IPV aggregation and denaturation, is linked to local mobilization of Eudragit E polymer chains. Then, the Eudragit E is believed to coat the virions and prevent aggregation by acting as an electrosteric stabilizer, increasing short-range steric repulsion due to its polymeric chain structure as well as long-range electrostatic repulsion among the virions via its positively charged side chains (45, 46).

Thus, when designing new formulations for IPV stabilization, polycations were examined that were similar to Eudragit E in their (*i*) effect on PLGA degradation and pH and (*ii*) effect on IPV stability at low pH. Polyethylenimine was identified as a candidate because of its high positive charge density and known buffering capacity in physiological ranges (47). In particular, bPEI was used because of its miscibility with organic solvents, which would allow it to associate closely with PLGA in the organic phase, and low molecular mass bPEI (1.8 kDa) was chosen to prevent toxicity (27). As expected, within the relevant pH range, bPEI had similar effects on PLGA degradation and buffering as Eudragit E. The slightly less pulsatile proton release profile from bPEI-containing particles (Fig. 1) and the higher concentration required to prevent IPV aggregation (*SI Appendix*, Fig. S1) may be because of the low molecular weight of the bPEI used, which could allow it to leach from PLGA particles over time, lowering its complexation efficiency and also decreasing the range of any steric stabilization effect it may have.

In contrast, PLL is water-soluble and has a history of use for electrostatic complexation and layering (26, 30, 48). It was therefore chosen for complexation with IPV virions to increase electrostatic repulsion under decreased pH conditions. Interestingly, all three of the polycations tested, Eudragit E, PLL, and bPEI, were better able to prevent type 3 IPV aggregation than type 1 aggregation (Fig. 2), likely because of the lower pI of type 3 IPV. Polycations may associate better with type 3 IPV than the other serotypes, explaining in part the greater improvement in type 3 IPV stability with PLL compared with the other serotypes (Fig. 4).

Because the F2 microsphere formulation, which used PLL as an IPV complexation agent and bPEI as a pH modulator, showed high release of all three IPV serotypes in D-antigen conformation (Fig. 5), the immunogenicity of F2 was tested in rats. As expected, the F2 microspheres elicited a stronger type 3 neutralizing response than F1 microspheres, with the seroconversion similar to that caused by the clinical control (2× bolus at *t* = 0 and *t* = 4 wk) and no statistically significant difference in absolute titers between the control and the F2 treatment group (Fig. 7). F2 microspheres were less effective than F1 in eliciting a neutralizing response to IPV types 1 and 2, although this difference was only statistically significant for type 2, in agreement with the only moderate effect of PLL and bPEI on type 1 and 2 stability measured in vitro (Fig. 5). Interestingly, while a single bolus injection of IPV elicits an IgG response, those antibodies cannot neutralize type 1 IPV without a second (boost) injection (Fig. 7 *A* and *D*). That a single injection of the F1 or F2 microspheres causes not only a stronger total IgG response, but also a strong neutralizing response suggests that the microsphere formulations elicit higher quality antibodies and not simply more antibodies, further demonstrating the utility of this strategy for vaccine delivery. Importantly, we did not observe any obvious local or systemic toxicity in response to the injection, which was as expected given that PLGA and similar polyesters have a long history of safety in the clinic (49–52).

Thus, PLGA can be used to encapsulate IPV, which, instead of only being thermostabilized with small molecule excipients, is also stabilized against pH changes using polycationic excipients like Eudragit E, PLL, and bPEI. The organic-soluble Eudragit E and bPEI interacted with PLGA, modulating the microsphere degradation kinetics while also buffering the internal microsphere environment and preventing a build-up of acidic degradation byproducts. In the hydrophilic compartment of the microspheres, all of the polycations tested, particularly PLL, mitigated pH-driven IPV aggregation that could lead to denaturation and may have additionally acted as a physical steric barrier to aggregation of nearby virions. Importantly, the acidic internal particle microenvironment may affect the ionization behavior of both the polycationic excipients and the IPV proteins: decreasing pH causes greater protonation and accumulation of positive charge on the polycations, thus reinforcing association of excipient with IPV and further improving electrosteric stabilization effects. In agreement with other reports (36, 37), we found that the three antigens in IPV, serotypes 1, 2, and 3 behaved very differently. In contrast to the work-intensive, empirical screening often required optimization small-molecule excipient formulations for biologics on a case-by-case basis (53, 54), our microparticle pH-neutralizing strategy using charged excipients of comparatively higher molecular weight appear to be broadly applicable to various antigens. Greater effects were seen for the IPV serotype with the most negative charge at neutral pH (type 3), suggesting that the virus particles and the polycations are associating via electrostatic interactions, but all three serotypes were positively affected by the polycations to some extent. Microspheres containing IPV and a combination of polycations that could both modulate PLGA degradation and also complex with IPV elicited a strong immune response in rats. To our knowledge, this report of a single-administration, pulsatile-release formulation of IPV that was able to achieve neutralizing antibody titers in vivo that were statistically equivalent to those achieved with a clinically relevant two-bolus control is unique. No adverse events were observed, nor would they be expected with this type of system, as PLGA microparticles have long been used successfully in the clinic, and IPV has not been causally associated with any serious adverse events.

Because vaccine stability in single-administration vaccines is critical for protection of the patient, a better understanding of the stabilizing excipients, including the polycations described here, will be crucial to designing successful formulations. This strategy of electrostatic complexation will potentially be applicable to other vaccine antigens whose stability is affected by acid-induced aggregation phenomena. As protein aggregation under various conditions, including in acidic media, is a common problem for long-term controlled release systems (6–9), this strategy has the potential for significant impact in the field of next-generation vaccines. This type of controlled release technology could serve as a platform for delivery of different types of vaccines with various vaccination schedules by simply altering the PLGA molecular weight or hydrophobicity to increase the time between bursts. Alternative strategies for the development of single-injection vaccines, including the recently reported SEAL technology (55), must also overcome similar challenges in vaccine stability and could benefit from the stabilization strategies described here. This technology can therefore serve as a tool to improve global health and aid in campaigns to control or eradicate infectious diseases, including polio.

## Materials and Methods

### Materials.

tIPV, composed of serotypes 1, 2, and 3 (Brünhilde strain type 1, MEF-1 strain type 2, and Saukett strain type 3 with 327 DU/mL, 70 DU/mL, and 279 DU/mL starting concentrations, respectively), were purchased from Statens Serum Institut (SSI) in their clinical formulations. For all experiments, one human dose of IPV was considered to be 40 DU, 8 DU, and 32 DU of types 1, 2, and 3, respectively, and the tIPV was used in this ratio. D-antigen content was determined using an ELISA kit from SSI. PLGA with an average molecular mass of 12 kDa and a lactide:glycolide ratio of 50:50, as well as Eudragit E PO, were purchased from Evonik. Gelatin from cold-water fish skin, maltodextrin (16.5–19.5 dextrose equivalent), PLL with a molecular mass range of 150–300 kDa, MSG, magnesium chloride hexahydrate (MgCl_2_), heavy mineral oil, and Span 80 were purchased from Sigma Aldrich. bPEI with a molecular mass of 1.8 kDa was purchased from Polysciences. All other materials were of at least reagent grade.

### Microsphere Formulations.

IPV and stabilizing excipients were coencapsulated in PLGA microspheres by the double emulsion method described previously (17). Briefly, tIPV was concentrated using an Amicon Ultracel centrifugal filter (Merck Millipore) with a molecular mass cut-off of 100 kDa and washed once with distilled water, yielding concentrated tIPV (IPV_conc_), with 148-, 150-, and 145-fold increases in the concentrations of type 1, 2, and 3, respectively, and with most of the excipients in the manufacturer’s initial formulation removed. The aqueous excipients were prepared as a solution of 20% maltodextrin, 17% MSG, and 17% MgCl_2_ in water. Aqueous excipients were mixed with IPV_conc_, with a final volumetric ratio of 2:1 (IPV_conc_:excipients) and a final ratio of 2.9:1 IPV doses to milligrams of excipient, forming the inner aqueous phase (*w*_*1*_). For microspheres containing PLL, a solution of PLL in 1× PBS was added to the IPV and mixed well before the final concentration and washing steps. For empty particle controls, water was used in place of IPV_conc_.

PLGA was dissolved in dichloromethane (DCM) along with organic excipients like Eudragit E and bPEI, forming the first oil phase (*o*_*1*_) with a total polymer concentration of 25 mg/mL. The first emulsion was formed by sonication of *w*_*1*_ in *o*_*1*_ in an ice bath for 20 s at 35% amplitude. The initial (theoretical) loading was 0.64 doses of IPV and 216 μg of aqueous excipients per milligram of polymer. The second emulsion was formed by adding heavy mineral oil with 3% Span 80 (*o*_*2*_) to the first emulsion at a 1:1 volumetric ratio of *o*_*1*_:*o*_*2*_ and vortexing at 3,500 rpm for 5 s. The *w*_*1*_/*o*_*1*_/*o*_*2*_ double emulsion was then poured into a stirring bath of heavy mineral oil with Span 80 for a final surfactant concentration of 0.4%.

The emulsion was stirred at 250 rpm at room temperature for 3 h to allow DCM evaporation. The hardened microspheres were then pelleted by centrifugation for 5 min at 3,200 × *g* at 4 °C. The excess oil and surfactant were decanted, and the pellet was washed three times by resuspension in hexanes and centrifugation at 200 × *g* for 3 min at 4 °C. After decanting the supernatant after the final wash, all residual hexanes and water were removed under vacuum for 1 h at room temperature. The dry particles were stored at 4 °C with dessicant until use.

### IPV Stability Measurements in Vitro.

The D-antigen content of IPV, as an in vitro correlate of protective immunogenicity, was measured by ELISA (SSI) according to the manufacturer’s instructions. Briefly, plates were coated with a monoclonal capture antibody specific to the D-antigen of either IPV serotype 1, 2, or 3 for 2–5 h at room temperature. The wells were washed using 1× PBS with 1% Triton-X, and samples and standards, diluted in the same buffer, were added for overnight incubation at 4 °C. The pH of all samples was determined before measurement by ELISA and, if necessary, was adjusted to a range of 6.5–8 to minimize interference with the assay. After washing excess samples and standards from the wells, an HRP-conjugated monoclonal detection antibody specific to the D-antigen of IPV serotype 1, 2, or 3 was diluted in 1× PBS with 50% FBS for blocking and added to the wells for incubation at room temperature for 1.5–3 h. The wells were washed again, and the IPV content in each well was detected using *o*-phenylenediamine dihydrochloride (OPD) reagent. Total IPV content, not specific to the D-antigen, was measured with an ELISA using polyclonal antibodies from rabbits immunized with denatured IPV by Spring Valley Laboratories, as described previously (17). IPV particle size was measured by DLS using a Wyatt Dyna Pro plate reader and by TEM using a JEOL 2100F TEM in the Nanotechnology Materials Core Facility at the David H. Koch Institute for Integrative Cancer Research.

To measure the effect of acidity on IPV physicochemical properties, tIPV was diluted fourfold with 1× PBS adjusted with 1 M hydrochloric acid (HCl) to pH 7.4, 6, or 4.5. Excipients were added to this solution at a final concentration of 2–100 µg/mL to test their stabilizing effects under these conditions. In the case of Eudragit E, the polymer was first dissolved in a solution of 0.7% HCl and 0.2% sodium chloride (NaCl) at 20 mg/mL, then diluted into the IPV solution. The pH of the final solution was verified in all cases to be unaffected by the addition of small amounts of excipients. These solutions were incubated at 37 °C with rotation. The condition of the IPV was then assessed by DLS and by ELISA.

### In Vitro Microsphere Characterization.

#### Microsphere size and morphology.

The size distribution of the microspheres was measured using a Multisizer 3 Coulter Counter (Beckman Coulter). For qualitative assessment of size and morphology, microspheres were mounted on conductive carbon tape, sputtered with gold, and imaged using a Jeol 5600LV SEM at the W. M. Keck Microscopy Facility at the Whitehead Institute for Biomedical Research.

#### Release kinetics.

For release studies, IPV-encapsulating microspheres were suspended in release buffer (1× PBS with 50 mM Hepes, 0.2% BSA, and 0.001% phenol red) at 10–15 mg/mL and incubated at 37 °C with rotation. At predetermined time points, the particles were pelleted by centrifugation at 1,500 × *g* for 5 min at 4 °C, and the full volume of the supernatant was removed and stored at 4 °C for no more than 1 wk before analysis. The same volume of fresh release medium was replaced in the tubes, and the particles were resuspended and returned to 37 °C with rotation until the next time point. Total IPV content and D-antigen content were measured by ELISA. The first burst or pulse of release was defined as the amount of IPV released over the first 4 d. The beginning of the second burst was defined as the first time point following the first burst at which more IPV was released than at the previous time point.

#### PLGA degradation and acidification.

At certain time points over the course of the release study, aliquots of microspheres were washed with water, frozen with liquid nitrogen, and lyophilized. The lyophilized particles were dissolved in tetrahydrofuran (THF), and the polymer/THF solution was filtered through a 0.2-µm poly(tetrafluoroethylene) (PTFE) syringe filter to remove particulates and insoluble proteins, sugars, and salts. The polymer was then measured by gel permeation chromatography to track the change in molecular weight over the course of the release study. Additionally, the pH of the supernatants collected during the release study was measured using a pH probe.

The efficacy of cationic excipients as pH modulators was assessed by measuring their buffering capacity. Five milligrams of each tested excipient was dissolved in 10 mL of 100 mM NaCl. The pH was adjusted to 11 using 1 M sodium hydroxide (NaOH) and then titrated to pH 3 using 0.1 M HCl. The number of protons buffered by the excipient was calculated relative to that of the 100 mM NaCl solution with no excipients.

### In Vivo Immunogenicity of IPV Formulations.

All procedures in animals were approved before beginning in vivo experiments by the Massachusetts Institute of Technology Committee on Animal Care. The immunogenicity of IPV microspheres formulations was tested in female Wistar rats, aged 8–12 wk at the start of the experiment. Rats were anesthetized by isoflurane inhalation and injected intramuscularly in the hind quadriceps, with 200 µL injected per site (400 μL total). To obtain serum samples for analysis, blood was collected from the lateral tail vein of rats, clotted, and centrifuged at 15,000 × *g* to separate the serum from the clot. Serum was frozen, and neutralizing antibody titers were measured for each sample by the Centers for Disease Control and Prevention. Animals with neutralizing titers >2^3^ were considered to have seroconverted. It should be noted that a titer of 2^10.5^, or 1,448.2, was the highest output value of the neutralizing assay used, and graphed values of 2^10.5^ should be considered to be 2^10.5^ or higher. Total binding IgG titers were measured by ELISA as previously reported (17).

All groups received the same total dose of D-antigen IPV over the course of the experiment (24 DU, 4.8 DU, and 19.2 DU of types 1, 2, and 3, respectively). Three control groups were tested using liquid tIPV diluted in 1× PBS: a single bolus administered at *t* = 0; a single bolus administered with empty particles at *t* = 0; and two boluses administered at *t* = 0 and *t* = 4 wk. Two experimental groups were tested: a single injection of F1 particles at *t* = 0; and a single injection of F2 particles at *t* = 0 (see Table 3 for formulations).

**Table 3.** IPV microsphere formulations used for rodent studies

Formulation	Aqueous excipients	Organic excipients
F1	8% maltodextrin	3% Eudragit E
	6.8% MSG	
	6.8% MgCl_2_	
F2	8% Maltodextrin	3% bPEI
	6.8% MSG	
	6.8% MgCl_2_	
	0.008% PLL (1:1 mol/mol PLL:IPV)	
Empty particles	8% Maltodextrin	3% Eudragit E
	6.8% MSG	
	6.8% MgCl_2_	

Percentages refer to the mass ratio of excipients to the PLGA microspheres.

### Statistics.

Unless otherwise indicated, results are reported as mean ± SD. For antibody titers, the normality of the log_2_(titer) values was verified by the D’Agostino and Pearson omnibus normality test using Prism 6 (GraphPad Software). Antibody titers are reported as geometric mean ± geometric SD, and a one-way ANOVA with a Dunnett posttest was used to determine statistically significant differences between experimental groups and the control. Differences were considered statistically significant when *P* < 0.05 (*) or very significant when *P* < 0.01 (**).

## Supplementary Material

Supplementary File
