# Supplementary material for: Stabilized single-injection inactivated polio vaccine elicits a strong neutralizing immune response
Source: Proc Natl Acad Sci U S A. 2018 May 21;115(23):E5269–78. doi: 10.1073/pnas.1720970115 (PMC6003376; doi:10.1073/pnas.1720970115)
Supplement: Supplementary File [file pnas.1720970115.sapp.pdf]

Supporting Materials

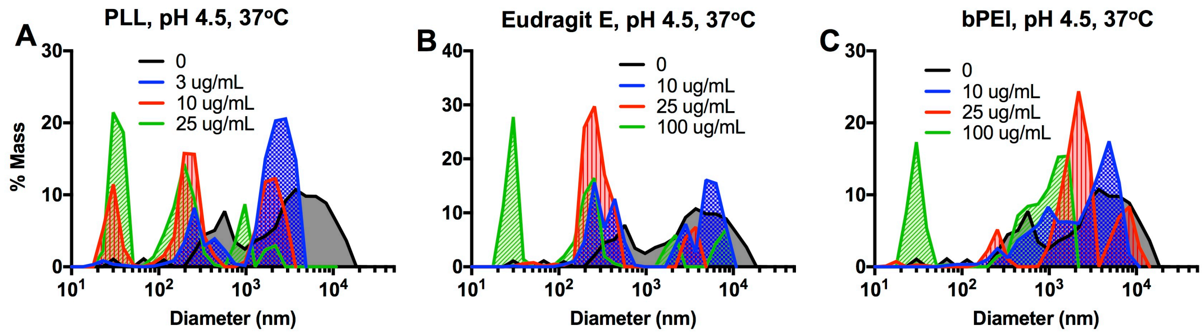

Supporting Figure S1. Polycations prevent acid-induced IPV aggregation in a concentration-dependent manner. Poly(L-lysine) (PLL), Eudragit E, and branched polyethylenimine (bPEI) prevent acid-induced IPV aggregation. This effect is concentration-dependent, and Eudragit E and bPEI both must be present in higher concentrations than PLL to prevent aggregation to the same extent.

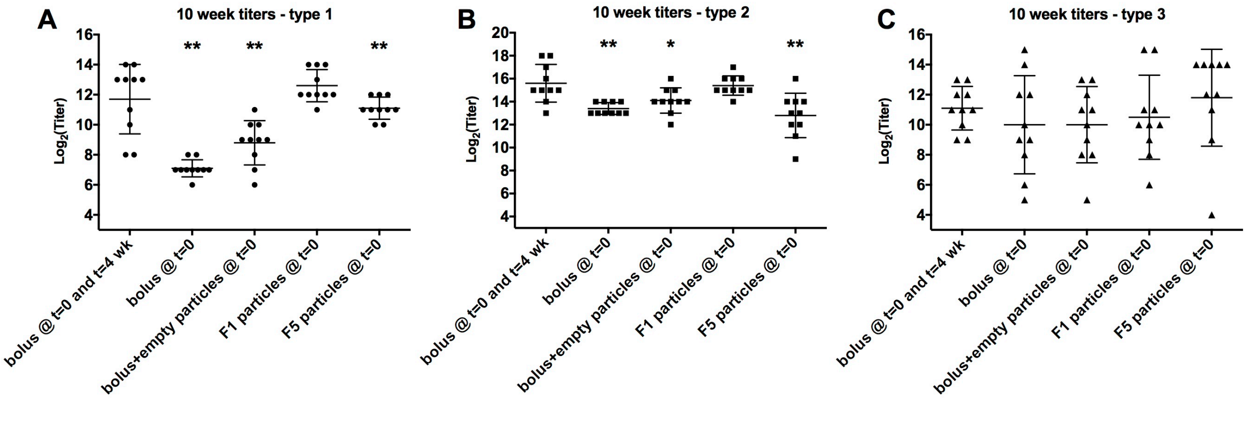

Supporting Figure S2. F1 or F5 particles elicit non-inferior total IgG titers at t=10 wk. At later time points, the total IgG titers elicited by F1 particles continue to be statistically equivalent to those elicited by the positive control (bolus injected at t=0 and t=4 wk) for types 1 and 2, while F5 particles are superior or statistically equivalent to the control from type 3. Asterisks indicate statistically significant differences (*p<0.05, **p<0.01) compared to the control (bolus injected at t=0 and t=4 wk).

Supporting Methods

Measurement of IPV-specific binding titers in immunized rats

After rats were injected with free IPV or IPV encapsulated in particles, the total IPV-specific binding IgG titers in the rat serum was measured over time. ELISA plates (96-well) were coated with monoclonal mouse anti-poliovirus antibodies from Thermo Scientific Pierce (Waltham, MA) diluted 1:1000 in 100 mM carbonate-bicarbonate buffer (pH 9.6) and incubated overnight at 4°C. The next day, the plates were washed three times with wash buffer (1×PBS + 0.05% Tween 20) and incubated for 1 hr at 37°C with blocking buffer (5% nonfat dry milk + 0.05% Tween 20 in 1×PBS). Then, this solution was replaced with 10 DU/mL IPV in blocking buffer and incubated at room temperature for 2 hr. The plates were washed three times with wash buffer, and a serial dilution (1:1) of serum samples in blocking buffer was added to the plates. After 2 hr incubation at 37°C, the plates were washed five times, and a polyclonal goat anti-rat IgG HRP-conjugated antibody (1:3000, Abcam, Cambridge, MA) diluted in blocking buffer was added to the wells. The plates were incubated 1 hr at 37°C. Finally, the plates were washed five times, and OPD peroxidase substrate (Sigma, St. Louis, MO) was added to the wells and allowed to develop for 30 min at room temperature. The reaction was stopped by adding 1 M H_2SO_4, and the absorbance of each well at 490 nm was read with a multiplate reader (Tecan). The titer reported is the last dilution whose absorbance was ≥ 2-fold higher than that of the negative control serum at the same dilution.

Production of polyclonal antibodies specific for denatured IPV

Monovalent IPV (types 1, 2, and 3 abbreviated as mIPV1, mIPV2, and mIPV3, respectively) was purchased from Serum Statens Institute (SSI). The antigens were individually denatured by heating to 60°C for 1 hr. Polyclonal antibodies were then produced from these

antigens by Spring Valley Laboratories (Woodbine, MD). For each serotype, two rabbits were immunized, and the serum, containing polyclonal antibodies specific for IPV, was recovered.

The serum was filtered through glass wool and a 0.2-μm filter. Antibodies were precipitated from the solution using 40% saturated ammonium sulfate (SAS). This mixture was stirred for 30 min, then centrifuged at 3000 rcf for 30 min at 4°C. The pellet was washed with 1×PBS with 40% SAS, centrifuged again, and dissolved in 1×PBS with 0.02% sodium azide. Small proteins were removed by filtration with an Amicon Ultracel-15 centrifugal filter with a 100 kDa MWCO. The retained proteins, including antibodies, were washed once with 1×PBS with 0.02% sodium azide, then centrifuged again. Then, the crude preparation of antibodies was desalted using a PD-10 column with Sephadex G-25 to remove all small molecules and salts other than PBS and sodium azide.

Two crude antibody preparations were obtained for each serotype, each produced by a different rabbit. For each serotype, one of these preparations was stored with 30% sterile glycerol at -80°C or -20°C for short-term storage and used as the coating antibody. The other preparation for each serotype was used as the detection antibody. For the detection antibody, the antibody isolation process was carried out without sodium azide. Then, the antibody preparation was mixed with 10-fold molar excess biotin sulfo-NHS (N-hydroxysuccinimide) for 1 hr at room temperature. Excess biotin was removed using a PD-10 column. The biotinylated detection antibodies were then stored with 30% sterile glycerol as described above.

Quantification of total IPV content by ELISA

The polyclonal antibodies against denatured IPV were used to detect total IPV content, non-specific to the D-antigen conformation. All assays were monovalent. For each, the coating antibody was diluted in 50 mM carbonate-bicarbonate buffer (pH 9.6) (type 1, 1:1000 dilution;

type 2, 1:2000; type 3, 1:500), and 50 μL of diluted antibody was added to each well of a 96-well plate. After incubation at 4°C overnight, the plates were washed with wash buffer (1×PBS + 1% Triton X-100). The wells were blocked with blocking buffer [1×PBS + 1% bovine serum albumin (BSA)] for 1 hr at room temperature. Then, monovalent IPV standards (from SSI) and all samples were denatured by heating to 60°C for 1 hr and then diluted in assay buffer (1×PBS + 1% BSA + 1% Triton X-100). The blocking buffer was removed, and 50 μL of each standard or sample was added to each well of the plate and incubated for 2 hr at room temperature. The plates were washed, and 50 μL of diluted detection (biotinylated) antibody was added to each well (type 1, 1:2000 dilution in assay buffer; type 2, 1:1000; type 3, 1:2000) for 2 hr at room temperature. The plates were washed, and 50 μL Extravidin-peroxidase (1:1000 dilution in assay buffer, Sigma, St. Louis, MO) was added to each well for 1 hr at room temperature. The plates were washed, and O-phenyldiamine dihydrochloride (OPD) substrate (Sigma) was added to the wells for peroxidase detection. The reaction was stopped after 30 min with 1 M sulfuric acid, and the absorbance at 490 nm was measured with a Tecan multiplate reader.
